# Validation of two portable bioelectrical impedance analyses for the assessment of body composition in school age children

**DOI:** 10.1371/journal.pone.0171568

**Published:** 2017-02-03

**Authors:** Li-Wen Lee, Yu-San Liao, Hsueh-Kuan Lu, Pei-Lin Hsiao, Yu-Yawn Chen, Ching-Chi Chi, Kuen-Chang Hsieh

**Affiliations:** 1 Department of Diagnostic Radiology, Chang Gung Memorial Hospital, Chiayi, Taiwan; 2 Department of Nursing, Chang Gung University of Science and Technology, Chiayi Campus, Chiayi, Taiwan; 3 Sport Science Research Center, National Taiwan University of Sport, Taichung, Taiwan; 4 Department of Physical Education, National Taiwan University of Sport, Taichung, Taiwan; 5 Department of Cosmetic Application and Management, St. Mary's Junior College of Medicine, Nursing and Management, Yilan, Taiwan; 6 Department of Dermatology, Chang Gung Memorial Hospital, Linkou, Taiwan; 7 Department of Dermatology, Chang Gung Memorial Hospital, Chiayi, Taiwan; 8 College of Medicine, Chang Gung University, Taoyuan, Taiwan; 9 Office of Physical Education and Sport, National Chung Hsing University, Taichung, Taiwan; 10 Research Center, Charder Electronic Co, Ltd, Taichung, Taiwan; University of Palermo, ITALY

## Abstract

**Background:**

Bioelectrical impedance analysis (BIA) is a convenient and child-friendly method for longitudinal analysis of changes in body composition. However, most validation studies of BIA have been performed on adult Caucasians. The present cross-sectional study investigated the validity of two portable BIA devices, the Inbody 230 (BIA_8MF_) and the Tanita BC-418 (BIA_8SF_), in healthy Taiwanese children.

**Methods:**

Children aged 7–12 years (72 boys and 78 girls) were recruited. Body composition was measured by the BIA_8SF_ and the BIA_8MF_. Dual X-ray absorptiometry (DXA) was used as the reference method.

**Results:**

There were strong linear correlations in body composition measurements between the BIA_8SF_ and DXA and between the BIA_8MF_ and DXA. Both BIAs underestimated fat mass (FM) and percentage body fat (%BF) relative to DXA in both genders The degree of agreement in lean body mass (LBM), FM, and %BF estimates was higher between BIA_8MF_ and DXA than between BIA_8SF_ and DXA. The Lin’s concordance correlation coefficient (ρ_c_) for LBM_8MF_ met the criteria of substantial to perfect agreement whereas the ρ_c_ for FM_8MF_ met the criteria of fair to substantial agreement. Bland-Altman analysis showed a clinically acceptable agreement between LBM measures by BIA_8MF_ and DXA. The limit of agreement in %BF estimation by BIA and DXA were wide and the errors were clinically important. For the estimation of ALM, BIA_8SF_ and BIA_8MF_ both provided poor accuracy.

**Conclusions:**

For all children, LBM measures were precise and accurate using the BIA_8MF_ whereas clinically significant errors occurred in FM and %BF estimates. Both BIAs underestimated FM and %BF in children. Thus, the body composition results obtained using the inbuilt equations of the BIA_8SF_ and BIA_8MF_ should be interpreted with caution, and high quality validation studies for specific subgroups of children are required prior to field research.

## Introduction

Growth monitoring is important for early detection of health and nutritional problems during child development. Growth charts of length-for-age, weight-for-age, and BMI-for-age are currently used to assess physical growth in children. These charts can provide a general clinical overview of the health and nutritional status of children. However, body composition undergoes dynamic changes throughout growth and development, and current growth charts only provide proxy measures for changes in body composition.

The techniques most commonly used to assess body composition in children are underwater weighing, isotope dilution, dual-energy X-ray absorptiometry (DXA), air-displaced plethysmography, and bioelectrical impedance analysis (BIA). Among these techniques, BIA employs portable equipment and is a safe, convenient, and child-friendly method that is suitable for measurement and tracking of body composition changes in children [1].

The two common BIA techniques are the whole-body and segmental modes, in which a current passes from hand-to-foot, foot-to-foot, or hand-to-hand, with subjects either in the supine position or standing [2]. Whole-body BIA employs four electrodes attached to different sides of the body for measurement of electrical resistance. Body composition parameters, such as fat free mass (FFM), lean body mass (LBM), fat mass (FM), and percentage body fat (%BF), are then calculated using specific equations based on recorded impedance, height, age, sex, anthropometric index, and other factors [3]. Multi-segmental BIA employs eight electrodes to calculate whole-body and regional body composition, and can provide information on the spatial distribution of different components of body composition and their changes over time [4]. Therefore, multi-segmental BIA is theoretically superior to classical BIA for studies of pediatric body composition. Moreover, multi-segmental BIA can provide an estimate of appendicular lean mass (ALM), which constitutes the majority of skeletal muscle mass (SM) and thus can be used as a proxy for SM [5, 6].

Multi-segmental BIA is available in single-frequency and multi-frequency modes. Single-frequency BIA generally employs a 50 kHz current that passes through extracellular and intracellular fluids for estimation of total body water [7]. The multi-frequency method uses multiple frequencies to differentiate intracellular from extracellular fluid, and, therefore, provides a better estimation of total body water than the single frequency method [7]. However, there is controversy concerning whether multi-frequency BIA provides more accurate estimates of body composition in children compared with the single frequency method [3, 8, 9].

Previous BIA validation studies were conducted predominantly in adult Caucasians [7]. Pietrobelli et al. [10] demonstrated that appendicular electrical resistance had a strong positive correlation with ALM in white healthy adults, and could be used to estimate the lean mass of the limbs. However, children are not simply “miniature adults”, thus, equations established for adults may not be applicable to children. Therefore, it is necessary to investigate the reliability and validity of different BIA devices before initiation of field studies on pediatric body composition.

This cross-sectional study of healthy Taiwanese children (age 7–12 years) examined the accuracy and validity of two portable multi-segmental BIA devices by comparing their results with those from DXA measurements.

## Materials and methods

### Study design

This cross-sectional study was approved by local Institutional Review Board of the Chang Gung Memorial Hospital (103-1027A3), and written informed consent was provided by the subjects and their parents. Subjects were recruited *via* hospital advertisements and word-of-mouth from February to December, 2015. All subjects were healthy Taiwanese children aged 7–12 years-old. None of the subjects were pregnant, had amputations, implants, or chronic illnesses, or were prescribed regular medication.

Participants were instructed to eat breakfast on the study day and then fasted completely for at least 2 h before reporting to the Chang Gung Memorial Hospital (Chiayi branch) between 8:30–11:00 am. Vigorous activities and alcohol were avoided for a minimum of 48 h before the study day. Girls were not given appointments during their menstrual cycle. On arrival, participants were asked to void and change into a hospital gown. All measurements including body weight, height, BIA, and DXA were completed on the same morning, with a total study time of approximately one hour. One measurement per subject was performed using each instrument. Body height (cm) and weight (kg) were measured with subjects wearing no shoes using a digital scale (Super-View, HW-3050, Taipei, Taiwan).

### Bioelectrical impedance analysis (BIA)

All BIA measurements were made by trained research assistants. Subjects were measured wearing hospital gowns (< 0.2 kg) and weight adjustment for clothing was not applied. A single-frequency (50 kHz, 500 μA) BIA device (Tanita BC-418, Tanita Corp., Tokyo, Japan), referred to as BIA_8SF_, was used to estimate LBM_8SF_, ALM_8SF_, FM_8SF_, and %BF_8SF_ [11]. This method allows bioelectricity impedance measurement of the whole body and each part (right leg, left leg, right arm and left arm). The age limits for the BIA_8SF_ are 7–99 years. After the sex, age and height information had been entered into the BIA_8SF_, subjects were asked to stand in a stable position with bare feet. Their toes and heels were placed in contact with the anterior and posterior electrodes of the weighting platform, respectively. The measurements began when the grips were grasped by both hands. With BIA_8SF_, electric current was supplied from the toe tips of both feet and the fingertips of both hands, and the voltage was measured on the heel of both feet and the thenar area of both hands. Finally, the inbuilt equation was used to convert the input impedance to body composition estimates. Test-retest reliability for whole body LBM and %BF estimates by BIA_8SF_ were both ≥ 0.99 (n = 5) using the intra-class correlation coefficient (ICC).

A multi-frequency (20 kHz and 100 kHz) BIA device using eight-point tactile electrode system (Inbody 230, Biospace Corp., Seoul, Korea), referred to as BIA_8MF_, was used to measure LBM_8MF_, ALM_8MF_, FM_8MF_, and %BF_8MF_ [12]. The BIA_8MF_ is suitable for individuals aged 3–99 years-old according to the manufacturer. The BIA_8MF_ produces 10 impedance values by using two different frequencies to measurement the five segments of the body (right leg, left leg, right arm, left arm and the trunk). The measurement procedure for BIA_8MF_ was similar to that for BIA_8SF_, except thumb should be placed on the electrode pad on the top surface of the handle for BIA_8MF_. Body composition estimates were calculated by using the manufacturer’s software (Lookin’Body 120, Biospace Corp., Seoul, Korea). Test-retest reliability for whole body LBM and %BF estimates by BIA_8MF_ were both ≥ 0.99 (n = 5) using ICC.

### Dual-energy X-ray absorptiometry (DXA)

DXA is the reference method for assessment of body composition. Whole body DXA was performed using a fan-beam system (Delphi A, QDR series, Hologic, Bedford, MA, USA) configured with software version 12.5. The scanner was equipped with switched pulse dual-energy x-ray tube, operating at 100 kVp and 140 kVp. The *in vivo* precision of the scanner for whole body measurement was 1.0%, according to the product specification. The scanner was calibrated daily with the Hologic spine and body composition step phantoms before scanning the subject. Then, subjects were instructed to lie supine on the scanning bed. The DXA operator manually assisted subjects to position within the scanning zone with their head, neck and torso parallel to the long-axis of the scanning bed; arms at their sides; palms down; legs internally rotated about 25° until the toes touched; and feet fixed together using strapping tape. Subjects were instructed to remain still and breathe normally during the scan. All DXA scans were analyzed by the same operator who followed the manufacturer’s instructions and used the pediatric mode and standardized cutoff for regional measurements [13]. The subregions were defined as the head, trunk, right arm, left arm, right leg, left leg. DXA measured regional and whole body composition, including LBM_DXA_, ALM_DXA_, FM_DXA_, and %BF_DXA_.

### Statistical analysis

The statistical software package SPSS version 17.0 (SPSS Inc., Chicago, IL, USA) was used for data analysis. All data are reported as means ± SDs. Analysis of variation (ANOVA) with Student’s independent *t*-test (two-sided) was applied for analysis of repeated measurements to compare the different testing methods. The statistical significance level was set at *α* = 0.05. Pearson’s product moment correlation and ordinary least products regression analysis were used to examine the relationship between the BIA and DXA and to determine the proportional bias and fixed bias [14]. The correlation coefficient (*r*) and determination coefficient (*r*^2^) from linear regression analysis were used to define the strength of linear association. The standard error of the estimate (SEE), a measure of the accuracy of predictions made with a linear regression, was used to assess the statistical conformity of the two BIA methods.

To assess the degree of agreement between BIA and DXA measurements, three statistical techniques were used: the ICC, Lin’s concordance correlation (CCC) and Bland-Altman plot. The ICC coefficient (*r*_1_) (with two-way random and single measure) was used to assess the agreement between BIA and DXA methods [15]. An *r*_1_ value ≥ 0.8 was considered a strong level of agreement. The CCC coefficient (ρ_c_) was used to assess how close the data from BIA and DXA methods was about the line of best fit and also how far that line was from the 45-degree line through the origin [16]. The ρ_c_ and a concordance scale used including ratings of almost perfect: ρ_c_ > 0.99; substantial: 0.99 ≥ ρ_c_ > 0.95; fair: 0.95 ≥ ρ_c_ ≥ 0.9; poor: ρ_c_ < 0.9) were used to assess the concordance of the two BIA methods [17]. Bland-Altman plot with a regression analysis using ordinary least squares regression was used to display the difference between a pair of measurements against the mean of the pair [18]. Limits of agreement (LOA) were used to assess the agreement between two readings obtained by BIA and DXA on the same variable.

## Results

A total of 150 children (72 boys and 78 girls) with a mean age of 9.3 ± 1.5 years were enrolled. Subject demographics and body composition estimates are shown in Table 1. There were no significant differences in age, height or weight between boys and girls. However, the boys had significantly higher BMI compared with the girls (18.3 ± 4.3 in boys and 17.1 ± 3.0 in girls, p = 0.038). Based on DXA results, FM and %BF showed no significant difference between boys and girls whereas the boys had significantly higher LBM and ALM than the girls. For both boys and girls, all body composition results by BIA_8MF_ and BIA_8SF_ were significantly different from the results by DXA (**P < 0.001, Table 1), except for LBM by BIA_8MF_.

**Table 1:** Anthropometric characteristics and body composition measurements of Taiwanese children (age 6 to 12 years) determined by DXA (reference method), BIA_8MF_, and BIA_8MF_.

	Boys (n = 72)			Girls (n = 78)			Total (n = 150)		
	Mean	SD	Range	Mean	SD	Range	Mean	SD	Range
Age (years)	9.4	1.6	7.1–12.7	9.2	1.5	7.1–12.1	9.3	1.5	7.1–12.7
Height (cm)	138.0	11.0	114.7–164.9	137.5	11.3	112.2–159.1	137.7	11.1	112.2–164.9
Weight (kg)	35.6	11.9	19.2–73.1	33.0	9.5	19.3–60.4	34.2	10.8	19.2–73.1
BMI	18.3*	4.3	13.4–30.0	17.1	3.0	12.3–26.6	17.7	3.7	12.2–30.0
LBM (kg)									
DXA	24.3	5.7	15.2–40.6	22.4	5.5	13.6–38.1	23.3	5.7	13.6–40.6
BIA_8MF_	24.1	5.7	14.9–39.0	22.8	5.6	13.9–38.6	23.4	5.7	13.9–39.0
BIA_8SF_	26.4**	5.3	17.2–39.6	24.8**	5.4	16.1–40.0	25.6**	5.4	16.1–40.0
FM (kg)									
DXA	10.9	7.6	3.6–35.7	10.2	4.9	4.3–24.7	10.6	6.3	3.6–35.7
BIA_8MF_	9.6**	7.2	2.8–34.6	8.5**	4.4	3.0–21.8	9.1**	5.9	2.8–34.6
BIA_8SF_	7.9**	7.6	1.1–35.2	6.9**	4.2	2.0–21.0	7.4**	6.1	1.1–35.2
%BF (%)									
DXA	27.3	10.3	13.4–48.2	29.2	7.1	17.7–47.6	28.3	8.8	13.4–48.2
BIA_8MF_	24.3**	10.5	11.6–47.2	24.7**	7.1	14.4–42.9	24.5**	8.8	11.6–47.2
BIA_8SF_	18.5**	12.6	4.7–48.0	19.5**	6.7	9.3–36.9	19.0**	10.0	4.7–48.0
ALM (kg)									
DXA	10.4	2.9	5.3–19.0	9.4	2.6	5.3–16.5	9.9	2.8	5.3–19.0
BIA_8MF_	13.3**	3.7	7.5–22.8	12.4**	3.5	6.8–22.5	12.9**	3.6	6.8–22.8
BIA_8SF_	12.3**	3.5	6.9–22.3	10.8**	2.5	7.0–18.4	11.5**	3.1	6.9–22.3

Abbreviations: ALM, appendicular lean mass; BIA_8SF_, Tanita BC-418; BIA_8MF_, Inbody 230; BMI, body mass index; DXA, dual-energy X-ray absorptiometry; FM, fat mass; LBM, lean body mass; SD, standard deviation; %BF: percent body fat.

*P < 0.05, by repeated-measures ANOVA with Student’s independent *t*-test;

**P < 0.01, by repeated-measures ANOVA with Student’s independent *t*-test

**Fig 1:**
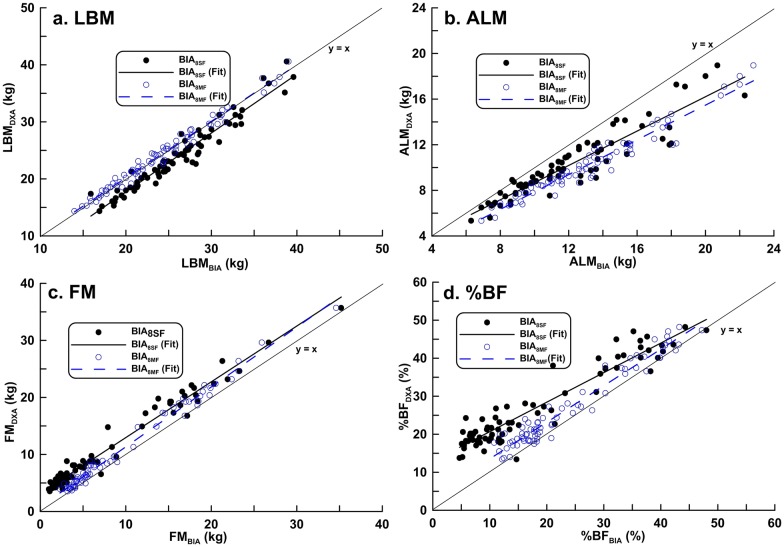
Correlation between dual-energy X-ray absorptiometry results and estimates of body composition in boys obtained with either BIA_8SF_ or BIA_8MF_. (a) LBM: BIA_8SF_: *r*^2^ = 0.940, BIA_8MF_: *r*^2^ = 0.979 (b) ALM: BIA_8SF_: *r*^2^ = 0.858, BIA_8MF_: *r*^2^ = 0.944 (c) FM: BIA_8SF_: *r*^2^ = 0.940, BIA_8MF_: *r*^2^ = 0.979 (d) %BF: BIA_8SF_: *r*^2^ = 0.898BIA_8MF_: *r*^2^ = 0.951.

**Fig 2:**
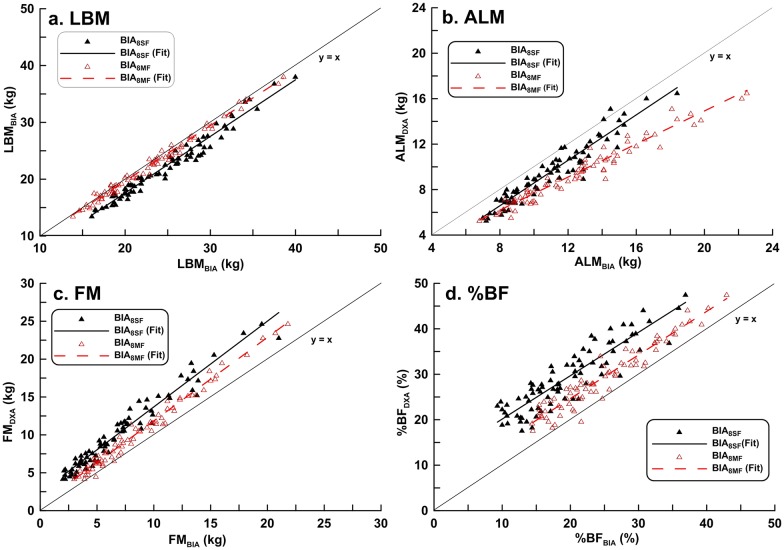
Correlation between dual-energy X-ray absorptiometry results and estimates of body composition in girls obtained with BIA_8SF_ or BIA_8MF_. (a) LBM: BIA_8SF_: *r*^2^ = 0.964, BIA_8MF_: *r*^2^ = 0.987 (b) ALM: BIA_8SF_: *r*^2^ = 0.915, BIA_8MF_: *r*^2^ = 0.951 (c) FM: BIA_8SF_: *r*^2^ = 0.953, BIA_8MF_: *r*^2^ = 0.981 (d) %BF: BIA_8SF_: *r*^2^ = 0.802, BIA_8MF_: *r*^2^ = 0.964.

Table 2 shows the Pearson product moment correlations coefficient (*r*) and the regression equation used to predict DXA results from BIA readings. There were strong linear correlations between the two BIA methods and DXA in the measurement of LBM, ALM, FM, and BF% (*r* ≥ 0.9 for all comparisons). However, there was a proportional bias and/or a fixed bias for each BIA measurement, except for LBM_8MF_. The scatter plots of body composition data by BIA and DXA methods showed BIA underestimated FM and %FM relative to DXA in both genders (Figs 1 and 2).

**Table 2:** Correlation of body composition estimates using Pearson product moment correlation and ordinary least products regression.

Method	*r*	a	95% CI	b	95% CI	Fixed bias	Proportional bias	SEE
Boys (*n* = 72)								
LBM_8SF_	0.971	-3.533	-5.188, -1.877	1.053	0.991, 1.115	Yes	No	1.368
LBM_8MF_	0.989	0.354	-0.509, 1.217	0.991	0.957, 1.026	No	No	0.839
FM_8SF_	0.986	3.248	2.813, 3.683	0.974	0.934, 1.014	Yes	No	1.283
FM_8MF_	0.993	0.854	0.508, 1.200	1.050	1.020, 1.078	Yes	Yes	0.876
%BF_8SF_	0.949	12.962	11.586, 14.339	0.776	0.715, 0.838	Yes	Yes	3.285
%BF_8MF_	0.976	3.880	2.530, 5.229	0.964	0.913, 1.014	Yes	No	2.256
ALM_8SF_	0.922	1.115	0.265, 2.178	0.748	0.673, 0.823	Yes	Yes	1.116
ALM_8MF_	0.970	0.287	-0.337, 0.912	0.758	0.713, 0.804	No	Yes	0.698
Girls (*n* = 78)								
LBM_8SF_	0.982	-2.354	-3.469, -1.329	0.996	0.952, 1.042	Yes	No	1.043
LBM_8MF_	0.994	0.213	-0.373, 0.800	0.972	0.947, 1.002	No	No	0.616
FM_8SF_	0.976	2.375	1.911, 2.840	1.132	1.074, 1.189	Yes	Yes	1.064
FM_8MF_	0.991	0.822	0.492, 1.153	1.102	1.068, 1.137	Yes	Yes	0.666
%BF_8SF_	0.897	10.609	8.407, 12.810	0.954	0.847, 1.061	Yes	No	3.141
%BF_8MF_	0.925	5.336	3.638, 7.434	0.984	0.892, 1.077	Yes	No	2.707
ALM_8SF_	0.956	-1.408	-2.190, -0.627	0.920	0.848, 0.989	Yes	Yes	0.783
ALM_8MF_	0.974	0.383	-0.110, 0.876	0.727	0.668, 0.765	No	Yes	0.596

Abbreviations: *r*, Pearson product moment correlation coefficient; a, b, coefficients in ordinary least products regression model: E(A) = a + b(B); *a*, (y axis) intercept; *b*, slope; fixed bias, if 95% confidence interval (CI) for a does not include 0; proportional bias, if 95% confidence interval (CI) for b does not include 1; SEE, standard error of the estimate.

Pearson correlation was used to quantify the strength of linear association between two methods of measuring the same variable, and it should not be used to assess agreement between methods. Therefore, the agreement of BIA_8SF_ and BIA_8MF_ with DXA was further examined using three statistical techniques: ICC, CCC and Bland-Altman plot (Table 3). In general, an ICC value (*r*_1_) ≥ 0.8 is considered a strong level of agreement. This study showed that all BIA parameters had *r*_1_ ≥ 0.9 except for LBM_8SF_ in boys, which was 0.887, indicating a strong agreement between the measures by BIA and DXA.

**Table 3:** Agreement between bioelectrical impedance analysis and dual-energy X-ray absorptiometry.

Method	Bland-Altman Plot				CCC (ρ_c_)	ICC (*r*_1_)
	Bias	Limit of agreement	Function	p		
Boys (*n* = 72)						
LBM_8SF_	2.12	-0.65 to 4.90	y = 0.082 x + 4.208	0.005	0.900	0.887
LBM_8MF_	-0.15	-1.82 to 1.52	y = -0.002 x − 0.096	0.902	0.989	0.943
FM_8SF_	-3.05	-5.63 to -0.47	y = 0.012 x − 3.156	0.571	0.911	0.973
FM_8MF_	-1.35	-3.21 to 0.55	y = -0.055 x − 0.763	< 0.0001	0.975	0.977
%BF_8SF_	-8.82%	-17.46 to -0.19%	y = 0.205 x − 13.526	< 0.0001	0.717	0.992
%BF_8MF_	-3.00%	-7.54 to 1.55%	y = 0.013 x − 3.335	0.620	0.936	0.986
ALM_8SF_	1.87	-0.97 to 4.71	y = 0.216 x − 0.585	< 0.0001	0.770	0.989
ALM_8MF_	2.93	0.69 to 5.17	y = 0.248 x + 0.016	< 0.0001	0.671	0.972
Girls (*n* = 78)						
LBM_8SF_	2.44	0.37 to 4.52	y = -0.015 x + 2.790	0.500	0.890	0.990
LBM_8MF_	0.37	-0.88 to 1.63	y = 0.018 x − 0.076	0.126	0.991	0.994
FM_8SF_	-3.29	-5.68 to -0.90	y = -0.150 x − 2.008	< 0.0001	0.763	0.992
FM_8MF_	-1.70	-3.30 to -0.10	y = -0.107 x − 0.691	< 0.0001	0.923	0.970
%BF_8SF_	-9.72%	-15.99 to -3.45%	y = -0.065 x − 8.149	0.229	0.445	0.989
%BF_8MF_	-4.48%	-8.50 to -0.46%	y = -0.001 x − 4.512	0.969	0.798	0.979
ALM_8SF_	1.42	-0.14 to 2.97	y = -0.045 x + 1.877	0.192	0.828	0.981
ALM_8MF_	3.01	0.74 to 5.28	y = 0.295 x − 0.208	< 0.0001	0.635	0.953

Abbreviation: CCC, Lin’s concordance correlation coefficient; ρ_c_, CCC coefficient; ICC, intra-class correlation; *r*_*1*_, ICC coefficient

In general, the CCC values (ρ_c_) for LBM, FM, and %BF were higher between BIA_8MF_ and DXA than between BIA_8SF_ and DXA (Table 3), indicating a better agreement between BIA_8MF_ and DXA measures. In both sexes, the ρ_c_ values for LBM, FM, and %BF were ≥ 0.9 between BIA_8MF_ and DXA, except for %BF_8MF_ in girls (Table 2). The ρ_c_ for LBM_8MF_ met the criteria for substantial to perfect agreement (ρ_c_ > 0.95) whereas the ρ_c_ for FM_8MF_ met the criteria for fair to substantial agreement (0.99 > ρ_c_ ≥ 0.9). For the %BF estimations, only the ρ_c_ values obtained by BIA_8MF_ in the boys (ρ_c_ = 0.936) met the criteria for fair agreement with DXA and the rest of the %BF estimations showed poor agreement (Table 3).

**Fig 3:**
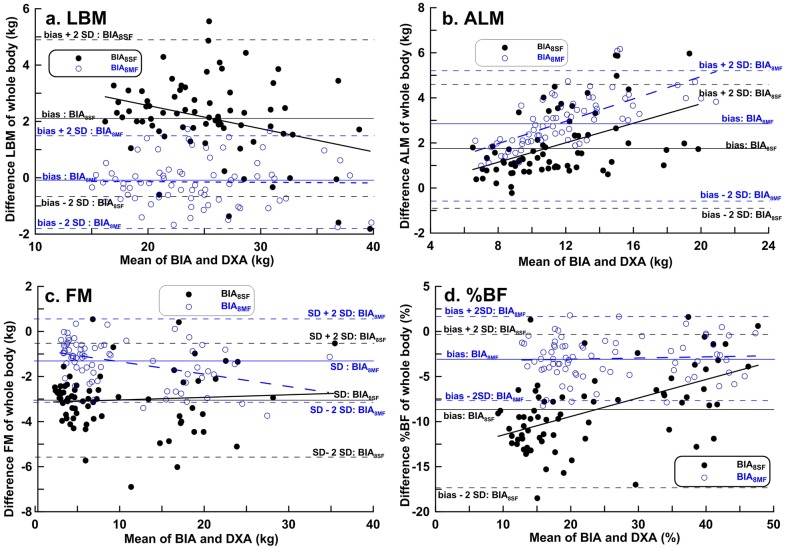
Bland-Altman plots with linear regression analysis of dual-energy X-ray absorptiometry results *vs*. BIA_8SF_ and BIA_8MF_ estimates of body composition in boys.

**Fig 4:**
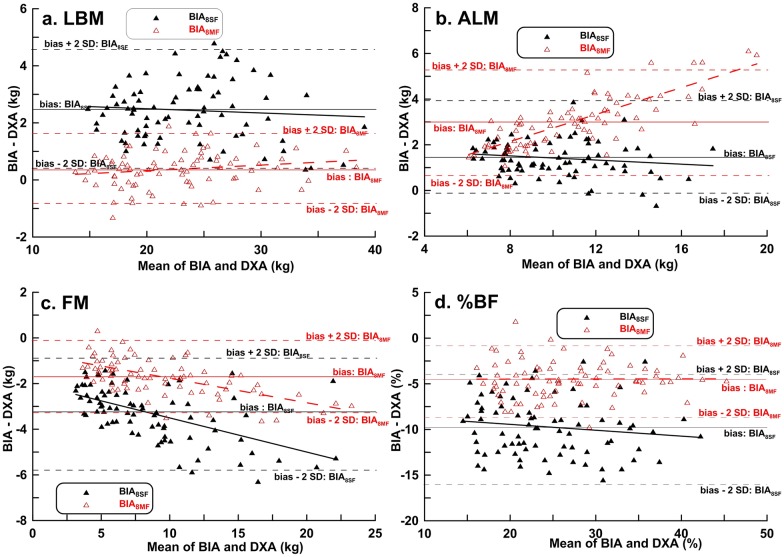
Bland-Altman plots with linear regression analysis of dual-energy X-ray absorptiometry results *vs*. BIA_8SF_ and BIA_8MF_ estimates of body composition in girls.

Bland-Altman plots were used to determine bias and LOA between BIA and DXA methods in boys (Fig 3) and girls (Fig 4). The LOAs were greater for the BIA_8SF_ and DXA measurements than for the BIA_8MF_ and DXA measurements, except for the ALM measures in girls (Table 3). Similar to the results by CCC, Bland-Altman analysis showed a good and clinically acceptable agreement between LBM measures by BIA_8MF_ and DXA (LOA = -1.82 to 1.52 kg in boys and LOA = -0.88 to1.63 kg in girls, Table 3).

In the human body, the FM is the total body weight minus LBM. Indeed, the LOAs of FM measures by BIA_8MF_ and DXA (-3.21 to 0.55 kg in boys and -3.30 to -0.10 kg in girls, Table 3) showed similar ranges to that of LBM but with different plus-minus sign (negative values in FM). In this study, the mean FM was about half of the LBM in children (Table 1) and thus, the degree of error was larger in FM estimation by BIA_8MF_ and DXA compared with that in LBM.

Regarding %BF estimation, BIA_8SF_ measurements underestimated %BF by 8.82% in boys and 9.72% in girls, whereas the BIA_8MF_ measurements underestimated %BF by 3.00% in boys and 4.48% in girls (Figs 3d and 4d). The LOAs in %BF estimation between BIA_8MF_ and DXA were clinically important. Even worse, there were larger LOAs in %BF estimation by BIA_8SF_ and DXA (-17.46 to -0.19% in boys and -15.99 to -3.45% in girls, Table 3**)**.

The ρ_c_ value for ALM estimated by BIA_8SF_ was 0.770 in boys and 0.828 in girls, and the ρ_c_ value for ALM estimated by BIA_8MF_ was 0.671 in boys and 0.635 in girls, all of which were considered poor agreement (Table 3). In agreement with CCC, Bland-Altman analysis showed a poor agreement with clinically importance between ALM estimations by BIA and DXA in both genders (Table 3).

## Discussion

This study compared the estimates of body composition obtained from multi-segment BIA_8SF_ and BIA_8MF_ with DXA measurements in primary school children from Taiwan. Pearson product moment correlation was used to test the linear association whereas ICC, CCC and Bland-Altman Plot were used to test agreement between BIA and DXA results. So far, there is still a debate about which method is the best for assessing agreement between two instruments. The ICC and CCC are scaled agreement indices depending on the measurement range, and therefore they are easy to summarize but hard to interpret [19]. In contrast, bias and LOAs (Bland-Altman plot) are unscaled indices based on the original unit and interpretation of the agreement requires prior knowledge of the measurement variables [20]. Since these methods all have some disadvantages, we have used more than one statistical method to assess agreement between two instruments in this study.

The LBM estimates by BIA_8MF_ and DXA were in high agreement for both genders using all statistical methods in this study. Therefore, BIA_8MF_ and DXA were interchangeable test methods for the measurement of LBM in children. However, the FM estimates showed fair to substantial agreement between BIA_8MF_ and DXA by CCC but clinically important differences by Bland-Altman plots. One possible explanation for the discrepancy in the degree of agreement may due to the fact that CCC was scaled relative to the between-subject variability and the large FM range in our subjects produced a relatively high ρ_c_ value. In contrast, Bland-Altman analysis was not dependent on between-subject variability such that it was easier to identify the error between the two methods.

Except for LBM estimates, the remainder of the BIA measurements showed strong linear correlated (but with clinically significant errors) with the gold standard method, DXA. Talma et al. [21] reported similar findings in a review article. Most previous BIA validation studies reported high precision using the BIA models but did not use a reference method to measure the accuracy of BIA estimates [22]. In addition to linear regression and ICC, we also performed Bland-Altman analysis and determined CCC to rigorously assess the statistical consistency of body composition estimates from BIA relative to DXA. Our results indicated clinically important errors in FM and %BF estimated by both BIA devices which may limit their applicability to body composition measurements at an individual level in children, even though the *r* and *r*_1_ values were high between both BIA methods and DXA. It is worth noting that although ICC is a popular test to compare the results between two methods, there is still a debate about the use of ICC in assessment agreement [23, 24].

We also compared both BIA_8SF_ and BIA_8MF_ models in children with a wide range of body fat composition, using DXA as the gold standard. Although the estimates from both BIA devices and DXA showed strong linear correlations, the correlation coefficients and agreements were higher for BIA_8MF_ compared with BIA_8SF_. In general, the BIA devices (especially the BIA_8SF_) overestimated LBM and underestimated FM. In addition, the LOAs were larger and the biases were greater for BIA_8SF_ measurements compared with BIA_8MF_ measurements, except for ALM in girls. The CCC analysis also indicated better agreements in measurements of LBM, FM, and %BF for the BIA_8MF_ in both sexes. These results confirm the findings of Kriemler et al. [25] that BIA_8MF_ is superior to BIA_8SF_ in pediatric body composition analysis.

In our study, both BIA_8SF_ and BIA_8MF_ underestimated FM and %BF in children who had large or small amounts of body fat. Additionally, BIA_8SF_ had a fixed bias or proportional bias in all components of body composition. Talma et al. [21], in their systematic review, indicated that BIA provided inconsistent results, depending on the reference method used. A literature review of validation studies for the Tanita BC-418 system in children also showed inconsistent results similar to our findings, whereas other studies had results which contradicted our findings. For example, Pietrobelli et al. [26] showed a perfect linear correlation between body composition parameters measured by the Tanita BC-418 system and DXA in subjects aged 6–64 years. However, they did not perform agreement analysis, and had a small sample size and wide age range. Some studies showed that the Tanita BC-418 underestimated FM in obese children compared with other reference methods [27, 28]. Shaikh et al. [28] reported a strong linear correlation between FM determined by the Tanita BC-418MA and DXA in obese boys aged 11.0 ± 0.53 years; however, the BIA system underestimated %BF, and the LOA in %BF was -3.8 to 15.4%. Haroun et al. [27] examined obese subjects (between 5–22 years of age) and found that the Tanita BC-418 underestimated FM by 3.5 kg in males and 3.6 kg in females, compared with the isotope dilution method. In contrast, Prins et al. [29] showed the Tanita BC-418MA system overestimated %BF in normal-weight Gambian children aged 5–16 years relative to the isotope dilution method.

We found that LBM estimates between BIA (BIA_8SF_ and BIA_8MF_) and DXA were in fair to substantial agreement whereas ALM estimates between BIA and DXA showed poor agreement. Few previous studies have used eight-electrode multi-frequency BIA devices (i.e. the Inbody-230) for estimates of body composition in children. Kriemler et al. [25] used a different BIA_8MF_ device (Inbody 3.0, Biospace, Seoul, Korea) in 6 years-old and found no fixed bias or proportional bias in FFM or ALM relative to measurements from DXA. Jensky-Squires et al. [30] used the Inbody-320 (Biospace, Seoul, Korea) to estimate %BF in children between 10–17 years of age relative to underwater weighing, and found significant differences in girls but not boys. Lim et al. [31] used the Inbody 720 (Biospace, Seoul, Korea) to estimate FFM, FM, and %BF in healthy children between 6–18 years of age and reported a high precision relative to DXA results. In their study, the LOA in %BF was -2.2 ± 6.1%, which was far less than ours.

BIA is primarily designed to estimate FFM, and the FFM prediction equations were developed using a reference method, such as DXA and/or isotope dilution. Variables in the regression equations may include height, weight, age, sex, race, and other factors [7]. Therefore, the established FFM equations may not applicable to all pediatric populations such as our pediatric populations [32, 33]. Body hydration status can also influence FFM calculation from BIA measurements. Most BIA prediction equations assume that the FFM consists of 73% water. However, although the water content of FFM is about 73% in adults, it is greater in children [22]. Therefore, a BIA prediction equation developed for adults could overestimate FFM in children. Moreover, hydration status changes as a child develops [34]. Therefore, an equation developed for school-aged children may not be accurate for adolescents. These major limitations of the BIA method remain unresolved.

## Conclusion

For all children, LBM measures using the BIA_8MF_ were precise and accurate whereas clinically significant errors occurred in both FM and %BF estimates. The BIA_8SF_ and BIA_8MF_ both underestimated FM and %BF in children. For the estimates of ALM, both BIA devices showed poor agreement with DXA. Thus, the body composition results obtained using the inbuilt equations of the BIA_8SF_ and BIA_8MF_ should be interpreted with caution, and high quality validation studies in specific subgroups children are required prior to field research.
